# Atypical Presentation of Hashimoto’s Disease in an Adolescent: Thyroid-Associated Ophthalmopathy

**DOI:** 10.4274/jcrpe.1450

**Published:** 2014-12-05

**Authors:** Heves Kırmızıbekmez, Rahime Gül Yeşiltepe Mutlu, Fatma Dursun, Murat Günay

**Affiliations:** 1 Zeynep Kamil Obstetrics and Children Education and Research Hospital, Clinic of Pediatric Endocrinology, İstanbul, Turkey; 2 Ümraniye Education and Research Hospital, Clinic of Pediatric Endocrinology, İstanbul, Turkey; 3 Zeynep Kamil Obstetrics and Children Education and Research Hospital, Clinic of Ophthalmology, İstanbul, Turkey

**Keywords:** Hashimoto’s disease, Graves’ disease, ophthalmopathy, children

## Abstract

Hashitoxicosis is generally differentiated from Graves’ hyperthyroidism by its shorter course and absence of ophthalmopathy. In this case report, we describe an adolescent girl who presented with significant clinical findings of hyperthyroidism, a diffuse goiter with homogenously increased uptake in scintigraphy, and with ocular findings of ophthalmopathy. The thyroid stimulating hormone receptor antibody test was positive, and the family history revealed thyroid-associated ophthalmopathy. Clinical findings supported the diagnosis of Hashimoto’s disease (HD) in the follow-up period. Radioactive iodine uptake investigation was found to be a reliable method for differential diagnosis. Attention was drawn to the rarity of pediatric cases of HD who present with ophthalmopathy.

## INTRODUCTION

Graves’ disease (GD) and Hashimoto’s disease (HD) are autoimmune thyroid diseases. GD is caused by stimulation of the thyroid-stimulating hormone (TSH) receptor located on the thyroid gland by an antibody known as the TSH-receptor antibody (TRAb). TRAb binds to the TSH receptor and stimulates the cyclic adenyl monophosphate signal transduction cascade and may lead to hyperplasia and hyperfunction of the thyroid gland (1). The cause of HD is thought to be a TSH stimulation-blocking antibody (TSBAb) which blocks the action of TSH leading to damage and atrophy of the thyroid gland (2).

HD is the most common form of thyroiditis in childhood, especially in iodine-replete areas of the world (3). A hyperthyroid metabolic state can be caused by the inflammation and destruction of thyroid follicles. Approximately 5 to 10 percent of children present with a thyrotoxic phase, a state sometimes termed “Hashitoxicosis”. Hashitoxicosis is generally differentiated from Graves’ hyperthyroidism by its shorter course, lasting a few months rather than years and by absence of ophthalmopathy (4). Measurement of TRAb is often used as a convenient, cost-effective method to confirm the diagnosis (5).

Thyroid-associated ophthalmopathy (TAO) is characterized by inflammation of the extraocular muscles and orbital fat/connective tissue, which results in proptosis, impairment of eye muscle function and periorbital edema (6). TRAb, as well as being a useful biomarker for disease activity, is also correlated with presence of ophthalmopathy independent of thyroid functions. TAO is a symptom typical for GD, but it very rarely accompanies HD (7).

## CASE REPORT

A thirteen-year-old girl complaining from a swelling on the anterior neck and bulging of the left eye was referred from another clinic for evaluation of suspected hyperthyroidism. She had a history of significant weight loss, anxiety and blurred vision for the last few weeks. Family history revealed thyroid disease which necessitated thyroidectomy in both paternal and maternal grandmothers and aunts. They were described as having toxic goiter with exophthalmos.

Physical examination revealed a patient with excessive sweating, mild tachycardia and diffuse goiter. Her TSH level was 0.004 mU/L, free thyroxine and triiodothyronine levels were elevated. The physical and laboratory findings of the patient are shown in Table 1. Methimazole and propranolol were prescribed. Anti- thyroid peroxidase and anti-thyroglobulin levels were very high, confirming autoimmune thyroiditis. TRAb test was positive, and Doppler ultrasonography demonstrated increased vascularisation of the thyroid gland. Radionuclide scanning with technetium-99m revealed a diffuse homogenous increased activity in the thyroid gland. Contrary to expectation, radioactive iodine uptake (RAI uptake) was found to be decreased. Ophthalmologic examination revealed lid retraction in the left eye.

Thyroid hormone levels normalized after 3 weeks and TSH normalized after five months of treatment. The patient was followed for 15 months, and the hyperthyroid state was corrected at month 5. Methimazole dose was tapered over two months and ceased. The thyroid gland was palpated to be of rubbery texture and its regression was observed in ultrasonography. The patient gained about 13 kilograms in five months. TRAb was found to be negative at 7-month follow-up. The same ophthalmologist who followed her reported a full recovery of the ophthalmopathy at month 5. The patient was found to be still euthyroid at the end of 12 months of follow-up.

## DISCUSSION

Our patient was an adolescent girl with hyperthyroidism who was thought to have GD based on the initial findings and who was treated with an anti-thyroid drug. Although RAI uptake was decreased, the clinical findings of hyperthyroidism, the presence of diffuse goiter with homogenously increased uptake in scintigraphy, presence of ophthalmopathy, positive family history for TAO and the positive TRAb test were findings which led us to continue the anti-thyroid treatment. The patient was followed for 15 months. Correction of the hyperthyroid state was achieved after 5 months of therapy, thus the anti-thyroid treatment was stopped. The ocular signs also disappeared; no relapse of hyperthyroidism occurred. Independent of thyroid hormone levels, the patient started to gain weight, possibly due to her increased appetite during the hyperthyroid phase. The clinical course of the patient supported the diagnosis of Hashitoxicosis and showed that RAI uptake investigation was a reliable method.

Hashitoxicosis is an uncommon but important cause of hyperthyroidism in children and has a variable clinical course. The diagnosis may be complicated, as the presenting features sometimes exhibit significant overlap with GD. Most authors assume HD and GD as concomitant states of chronic thyroiditis and consider both diseases as two sides of the same coin (8). Nabhan et al (9) investigated 69 pediatric patients and could not identify any predisposing factor for the development of Hashitoxicosis. The duration of hyperthyroidism in these cases was reported to range from 1 to 23 months and to be positively correlated with thyroid peroxidase autoantibody levels at presentation. In the few patients with more severe presentation, methimazole treatment was required (9,10).

TRAb is typically negative in patients with Hashitoxicosis. Some patients produce both thyroid-stimulating antibodies and thyroid-blocking antibodies. The clinical course depends on which antibody is predominant. A recent multicenter collaborative approach assessed the relevance of thyroid-stimulating antibodies in a large international pediatric group and reported no positive TRAb in patients with HD and a positive TRAb in most patients with GD. Antibody levels were found to positively correlate with the presence of ophthalmopathy (11). Approximately 50 to 75 percent of children with GD have some features of Graves’ ophthalmopathy and the symptoms tend to be milder than those seen in adults. Although TAO is almost always caused by GD, it is also said to occur in 5% of patients with HD. The role of cytotoxic T-lymphocytes, which cause the destruction of thyroid cells in HD, should not be underestimated (7). TAO is heterogeneous either due to different phase of presentation or different pathogenesis in different patients (6).

A few children and adolescents suffer from thyrotoxicosis during the course of autoimmune thyroiditis. In any case, it is apparent that infiltrative ophtalmopathy is very rare in children and adolescents. According to a literature survey covering several case reports and series of children with hyperthyroidism, especially GD, presentation with ophthalmopathy was not mentioned in any pediatric patient with Hashitoxicosis or with hyperthyroidism due to other etiologies (12). Hoping to contribute to the current knowledge on this rare condition, we herein presented this case of autoimmune thyroiditis in an adolescent female patient.

## Figures and Tables

**Table 1:**
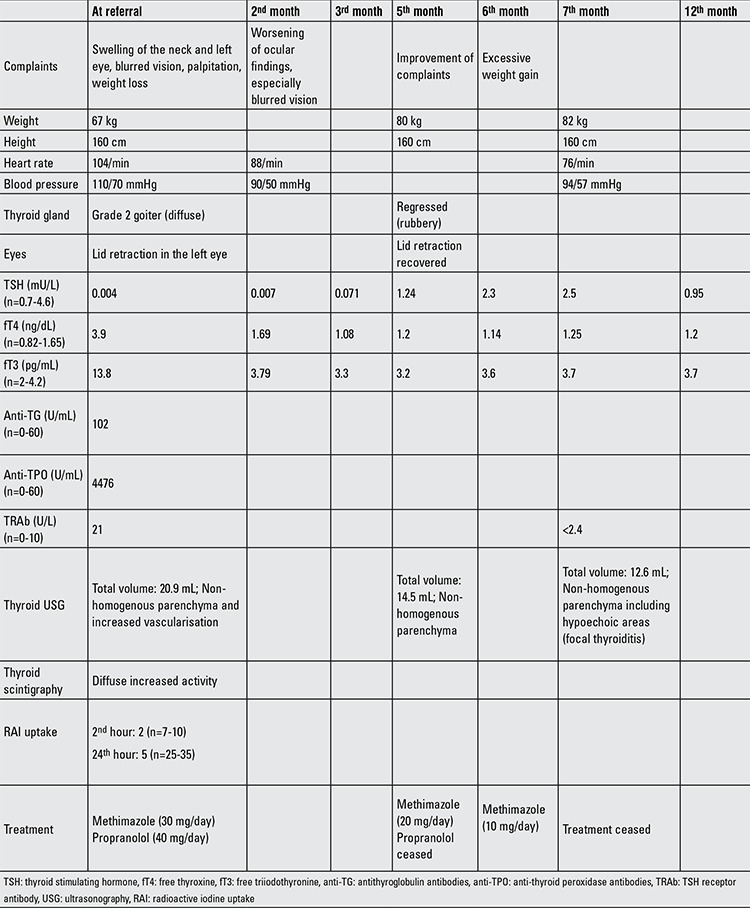
Clinical and laboratory findings of the patient at referral and at follow-up
